# Twiddler’s Syndrome

**DOI:** 10.5811/cpcem.2019.4.42123

**Published:** 2019-05-20

**Authors:** Jason A. Lesnick, Benjamin L. Cooper, Pratik B. Doshi

**Affiliations:** McGovern Medical School at the University of Texas Health Science Center in Houston (UTHealth), Department of Emergency Medicine, Houston, Texas

## Abstract

Twiddler’s syndrome refers to a rare condition in which a pacemaker or automatic implantable cardioverter-defibrillator (AICD) malfunctions due to coiling of the device in the skin pocket and resultant lead displacement. This image is the chest radiograph (CXR) of a 54-year-old male who presented to the emergency department with chest pain five months after his AICD was placed. The CXR shows AICD leads coiled around the device and the absence of leads in the ventricle consistent with Twiddler’s syndrome. Patients with twiddler’s syndrome should be admitted for operative intervention.

## CASE PRESENTATION

A 54-year-old male with an automatic implantable cardioverter-defibrillator (AICD) placed five months prior to arrival presented with sharp, left-sided chest pain for one day. He stated that his “pacemaker is moving.” On physical exam, the vital signs were within normal limits, the patient was in no distress, and the left superolateral chest wall was tender to palpation. The electrocardiogram showed a normal sinus rhythm without ischemic changes. Chest radiograph revealed AICD leads wrapped around the device and absence of leads in the ventricle (Image).

## DIAGNOSIS

Twiddler’s syndrome refers to pacemaker or AICD malfunction due to coiling of the device in the skin pocket and resultant lead displacement. It is rare, estimated to occur in 0.07 – 7% of implanted devices, and almost always occurs within the first year of implantation.^1^ It requires urgent attention as patients with malfunctioning AICDs are at risk for ventricular dysrhythmias and death,^2^,^3^ and patients who rely on pacemakers lose extrinsic pacemaking activity. Patients with twiddler’s syndrome should be admitted to a telemetry bed for operative repair. Twiddler’s syndrome is classically associated with the patient twiddling or twisting his pacemaker causing lead dislodgement. While our patient denied intentionally manipulating the device, he did mention that he felt like his pacemaker moved when he changed positions.

As implantable devices increase in popularity, emergency physicians should be aware of this potentially life-threatening condition.

**CPC-EM Capsule**

What do we already know about this clinical entity?

Twiddler’s syndrome is a rare condition in which a pacemaker malfunctions due to coiling of the device in the skin pocket and resultant lead displacement.

What is the major impact of the image(s)?

The chest radiograph shows AICD leads coiled around the device and the absence of leads in the ventricle consistent with twiddler’s syndrome.

How might this improve emergency medicine practice?

Patients with twiddler’s syndrome should be admitted for operative intervention.

## Figures and Tables

**Image:**
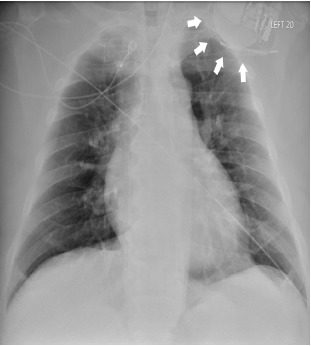
Anteroposterior chest radiograph revealing automatic implantable cardioverter-defibrillator leads wrapped around the device (white arrows) and absence of leads in the ventricle.
